# Hydrazide–hydrazones as potential antimicrobial agents: overview of the literature since 2010

**DOI:** 10.1007/s00044-016-1756-y

**Published:** 2016-11-25

**Authors:** Łukasz Popiołek

**Affiliations:** Department of Organic Chemistry, Faculty of Pharmacy, Medical University of Lublin, 4A Chodźki Street, Lublin, 20-093 Poland

**Keywords:** Hydrazide–hydrazone, Antibacterial activity, Antitubercular activity, Antifungal activity, MIC

## Abstract

Hydrazide–hydrazone derivatives are present in many bioactive molecules and display a wide variety of biological activities, such as antibacterial, antitubercular, antifungal, anticancer, anti-inflammatory, anticonvulsant, antiviral, and antiprotozoal action. Therefore, many medicinal chemists synthesize various hydrazide–hydrazones and evaluate them for biological activities. Among biological properties of this class of compounds, antimicrobial activity is the most frequently encountered in scientific literature. This paper is focused on the overview of the literature findings of the last six years (2010–2016) covering the research on antimicrobial activity of hydrazide–hydrazone derivatives. This review may also serve as a useful guide for the development of new hydrazide–hydrazones as potential antimicrobial agents.

## Introduction

Hydrazide–hydrazones constitute a class of organic compounds, which attracts the attention of medicinal chemists due to the fact that they contain azomethine group (–NH–N=CH–) connected with carbonyl group, which is responsible for their different pharmaceutical applications and makes possible the synthesis of different heterocyclic scaffolds (Rollas and Küçükgüzel 2007), like 1,3,4-oxadiazolines (Doğan et al. 1998), azetidin-2-ones (Kalsi et al. 2006), coumarins (Mohareb et al. 2011), 1,3-thiazolidin-4-ones (Popiołek et al. 2015, 2016a), and 1,3-benzothiazin-4-ones (Popiołek et al*.* 2016b).

The main route to synthesize hydrazide–hydrazone derivatives is the heating of appropriate hydrazides of carboxylic or heterocarboxylic acids with different aldehydes or ketones in various organic solvents like ethanol, methanol or butanol (Bala et al. 2013; Popiołek et al*.* 2015, 2016a; Popiołek and Biernasiuk 2016a, b). The molecular stucture of synthesized hydrazide–hydrazone derivatives can be easily confirmed by spectral methods. In the IR spectra, three characteristic bands are observed. The peaks around 1550 cm^−1^ correspond to the presence of C=N group. Carbonyl group (C=O) gives a characteristic band around 1650 cm^−1^, whereas the NH group can be found in the area around 3050 cm^−1^. In the ^1^H NMR spectra of hydrazide–hydrazones, we can observe a characteristic singlet signal in the range of *δ* 8–9 ppm, and the second singlet signal around *δ* 10–13 ppm, which correspond to =CH and NH groups, respectively. In the ^13^C NMR spectra, the signal for =CH group usually appears around *δ* 145–160 ppm, whereas in the range of *δ* 160–170 ppm we can observe the signal for carbonyl group (C=O) (Mohareb et al. 2011, Popiołek and Biernasiuk 2016a, b).

**Fig. 1:**
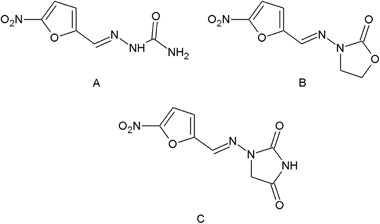
Chemical structures of medicines containing hydrazide–hydrazone moiety: nitrofurazone (**a**), furazolidone (**b**) and nitrofurantoin (**c**)

In recent years, a lot of biologically important hydrazide–hydrazone derivatives with a number of functional groups have been synthesized from many different carbonyl compounds. They were found to possess anticancer (Kumar et al. 2012; Yadagiri et al. 2014; Machakanur et al. 2012; Nasr et al. 2014), anti-inflammatory (Kumar et al. 2015), anticonvulsant (Çakır et al. 2001), antiviral (Şenkardes et al*.* 2016), and antiprotozoal (Siddiqui et al*.* 2014) activities. Among the biological properties of this class of compounds, the antimicrobial activity is the most frequently encountered one in scientific literature. Additionally, widely used chemotherapeutic agents such as nitrofurazone (McCalla et al. 1970), furazolidone (Chatterjee and Ghosh 1979; Ali 1983), and nitrofurantoin (McOsker and Fitzpatrick 1994; Munoz-Davila 2014) are known to contain typical hydrazide–hydrazone moiety or hydrazide-hydrazone moiety, in which the carbonyl group and nitrogen atom are included in the 1,3-oxazolidine-2-one or imidazolidine-2,4-dione ring (Fig. 1).

Encouraged by the above mentioned facts, this study is an attempt to collect the hydrazide–hydrazone derivatives, which can be considered as potential antimicrobial agents, reported in the literature in the years 2010–2016.

### Antibacterial activity

Searching for effective and non-toxic chemotherapeutic agents is still a very important issue due to the increase of multi-resistant bacterial strains (Moellering 2011). The treatment of bacterial infections is especially challenging in patients with compromised immune systems or with other associated diseases (Coates et al. 2002). Some of currently used antibacterial agents are known to contain hydrazide–hydrazone moiety (McCalla et al. 1970; Chatterjee and Ghosh 1979; Ali 1983; McOsker and Fitzpatrick 1994; Munoz-Davila 2014) (Fig. 1). Due to this fact, it is reasonable to search for novel antibacterial agents among hydrazide–hydrazone derivatives.

**Fig. 2:**
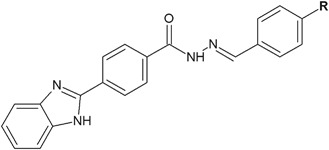
Benzimidazoles showing interesting activity against *Salmonella typhimurium.* **R** = Cl (**1**); Br (**2**)

The in vitro screening results of newly synthesized benzimidazole derivatives bearing hydrazone moiety revealed that some of the compounds had significant antimicrobial activity (Özkay et al. 2010). Among synthesized derivatives, compounds **1** and **2** had bactericidal effect on the growth of *Salmonella typhimurium*, two times better (compound **1**: MIC = 6.25 μg/ml) or equal (compound **2**: MIC = 12.5 μg/ml) to the activity of chloramphenicol (MIC = 12.5 μg/ml), which was used as positive control (Fig. 2). The activity of these compounds against other Gram-negative bacterial strains, like *Escherichia coli, Proteus vulgaris, Klebsiella pneumoniae*, or *Pseudomonas aeruginosa*, was good (MIC = 25–100 μg/ml). The activity against Gram-positive bacteria was assessed on four strains: *Listeria monocytogenes*, *Staphylococcus aureus*, *Enterococcus faecalis*, and *Bacillus subtilis*. The best activity equal to the activity of chloramphenicol was found against *E. faecalis* (MIC = 12.5 μg/ml). Against other Gram-positive bacterial strains, the activity of compounds **1** and **2** was good to moderate (MIC = 25–200 μg/ml) (Özkay et al. 2010).

**Fig. 3:**
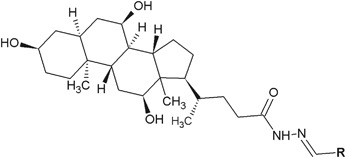
New derivatives of cholic acid with hydrazide–hydrazone moiety. **R** = 4-ClC_6_H_4_ (**3**); 4-BrC_6_H_4_ (**4**); 4-NO_2_C_6_H_4_ (**5**); 3-ClC_6_H_4_ (**6**); 4-Cl-3-NO_2_C_6_H_3_ (**7**)

In another research, Rasras et al*.* (2010) synthesized novel hydrazide–hydrazones of cholic acid and tested them for antibacterial activity against three Gram-negative and three Gram-positive bacterial strains (Fig. 3). The activity of five derivatives (**3**–**7**) against *E. coli* was strong (MIC = 3.91–7.81 µg/ml), but weaker than cefixime, which was used as control. Interestingly, none of the tested compounds had any activity against *P. aeruginosa* and *Enterobacter aerogenes*. In turn, the activity against Gram-positive bacterium *Enterococcus faecalis* for the compounds **3** and **6** (MIC = 1.96 µg/ml), **4** and **7** (MIC = 3.91 µg/ml), and **5** (MIC = 7.82 µg/ml) was almost 16, 8, and 4 times higher, respectively, than the activity of cefaclor (MIC = 31.25 µg/ml) and cefixime (MIC = 31.25 µg/ml). The MIC values for tested compounds against two other Gram-positive bacteria *Staphyloccocus aureus* and *Bacillus megaterium* were also good (MIC = 7.82–62.5 µg/ml) and comparable to chemotherapeutics used as controls (Table 1) (Rasras et al*.* 2010).

**Table 1:** In vitro antibacterial screening results of novel hydrazide-hydrazones of cholic acid

No. of compound	R	MIC (μg/ml)			
		Gram-negative bacteria	Gram-positive bacteria		
		*E. coli*	*S. aureus*	*E. faecalis*	*B. megaterium*
**3**	4-ClC_6_H_4_	3.91	62.5	1.96	31.25
**4**	4-BrC_6_H_4_	3.91	62.5	3.91	7.82
**5**	4-NO_2_C_6_H_4_	7.81	31.25	7.82	31.25
**6**	3-ClC_6_H_4_	3.91	31.25	1.96	31.25
**7**	4-Cl-3-NO_2_C_6_H_3_	7.81	31.25	3.91	31.25
Cefaclor	–	na	31.25	31.25	31.25
Cefixime	–	1.96	31.25	31.25	na

*na* not active, – not applicable

**Fig. 4:**
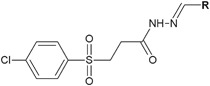
Hydrazide–hydrazones of 4-chlorophenylsulfonyl acid with antibacterial activity. **R** = 3-OCH_3_-C_6_H_4_ (**8**); 3-OH-C_6_H_4_ (**9**); 4-OH-3-OCH_3_-C_6_H_3_ (**10**)

Kumar et al. (2011a) evaluated novel hydrazide–hydrazones of 4-chlorophenylsulfonyl acid for in vitro antibacterial activity. The tested compounds (**8**, **9** and **10**) showed moderate to mild antibacterial activity on the basis of the measurement of the zone of the inhibition growth (ZOI = 10–21 mm) against two Gram-positive (*Bacillus subtillis* and *S. aureus*) and two Gram-negative (*E. coli* and *Salmonella typhi*) bacterial strains, when compared to ampicillin sodium used as a control (ZOI = 20–24 mm) (Fig. 4). The best antibacterial activity was displayed by compound **8** (ZOI = 21 mm) against *S. aureus* (control ZOI = 22 mm) and compound **10** against *E. coli*, whose zone of inhibition was 21 mm, whereas for ampicillin it was 20 mm (Table 2.) (Kumar et al. 2011a).

**Table 2:** Results of in vitro antibacterial assays of 4-chlorophenylsulfonyl acid derivatives

No. of compound	R	Zone of inhibition growth (mm)					
		Gram-positive bacteria		Gram-negative bacteria		Fungi	
		*B. subtilis*	*S. aureus*	*E. coli*	*S. typhi*	*C. albicans*	*A. niger*
**8**	3-OCH_3_-C_6_H_4_	19	21	10	10	31	20
**9**	3-OH-C_6_H_4_	17	20	11	14	26	21
**10**	4-OH-3-OCH_3_-C_6_H_3_	19	16	21	19	26	18
Ampicillin sodium	–	24	22	20	21	–	–
Clotrimazole	–	–	–	–	–	30	22

– not applicable

**Fig. 5:**
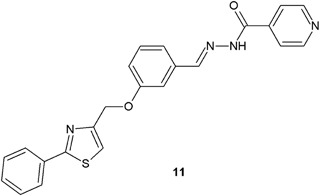
Isonicotinoyl hydrazide analog with significant activity against *S. aureus*

Among the isonicotinoyl hydrazide analogs synthesized by Moldovan et al. (2011), compound **11** appeared to have the strongest antibacterial activity. This hydrazide–hydrazone derivative possesses similar to ampicillin (ZOI = 16 mm) antibacterial activity (ZOI = 15 mm) against *S. aureus* (Fig. 5). The activity against other Gram-positive (*Bacillus cereus*) and Gram-negative bacteria (*E. coli*, *Salmonella thyphimurium*, *Proteus mirabilis*, and *Salmonella enterica*) was good to moderate (ZOI = 12–17 mm), when compared with chemotherapeutics used as controls: ampicillin, ciprofloxacin, gentamicin, and co-trimoxazole (Moldovan et al. 2011).

**Fig. 6:**
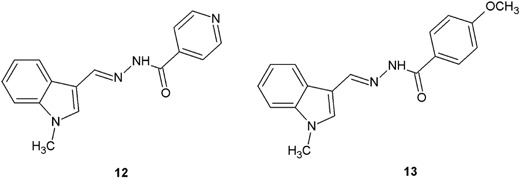
Indoles containing hydrazide–hydrazone moiety

In another research, indoles containing hydrazide–hydrazone moiety (**12**, **13**) synthesized by Shirinzadeh et al*.* (2011), showed good to moderate activity (MIC = 50–100 μg/ml) against the tested bacterial strains (Fig. 6).

**Fig. 7:**
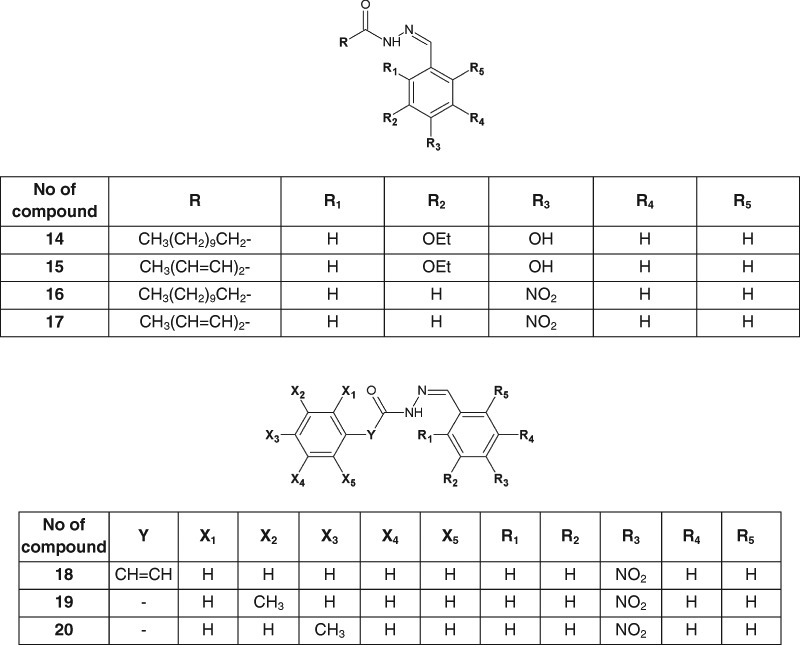
New 3-ethoxy-4-hydroxybenzylidene/4-nitrobenzylidene hydrazide–hydrazones with significant antibacterial activity

Kumar et al. (2011b) synthesized and evaluated antimicrobial assays, as well as performed QSAR studies of twenty 3-ethoxy-4-hydroxybenzylidene/4-nitrobenzylidene hydrazides. Seven of the new compounds (**14**–**20**) showed the antibacterial activity higher than that of ciprofloxacin against *S. aureus*, *B. subtilis*, and *E. coli* (Fig. 7) (Kumar et al. 2011b).

**Fig. 8:**
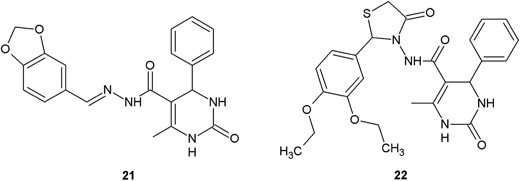
1,2-Dihydropyrimidine derivatives with antibacterial activity

Two derivatives of 1,2-dihydropyrimidine (**21**, **22**) synthesized by Al-Sharifi and Patel (2012) showed significant antibacterial activity against a panel of Gram-positive bacteria, including *B. subtilis*, *S. aureus* and, *Micrococcus luteus*, and Gram-negative bacteria, like *E. coli* and *Pseudomonas picketti* (Fig. 8) MIC values against these bacterial strains were in the range of 0.08–1 µg/ml, which can be assessed as very strong antibacterial activity. It is worth to underline that the lowest value of MIC was presented by compound **21** against *M. luteus* (MIC = 0.08 µg/ml) (Al-Sharifi and Patel 2012).

**Fig. 9:**
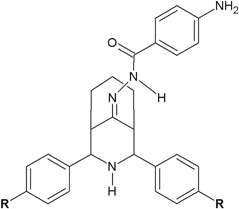
Novel hydrazide–hydrazones obtained from 4-aminobenzoic acid hydrazide. **R** = Br (**23**); Cl (**24**); OCH_3_ (**25**)

Xaiver et al*.* (2012) synthesized novel hydrazide–hydrazones as a result of condensation of 2,4-diaryl-3-azabicyclo[3.3.1]nonan-9-ones with 4-aminobenzoic acid hydrazide (Fig. 9). The obtained compounds were tested for in vitro antibacterial activity against eight bacterial strains (Gram negative bacteria: *S. thypimurium*, *E. coli*, *Vibrio cholerae*, *S. typhi*, *P. aeruginosa*, and *K. pneumonia*, and Gram-positive bacteria: *B. subtilis* and *S. aureus*). Among the synthesized derivatives, it is worth to mention two compounds **23**, **24**, which showed good to moderate activity against all bacterial strains (MIC = 50–200 μg/ml) (Table 3) (Xaiver et al*.* 2012).

**Table 3:** In vitro antibacterial screening results of 4-aminobenzoic acid hydrazide derivatives

No. of compound	R	MIC (μg/ml)							
		Gram-negative bacteria						Gram-positive bacteria	
		*S. typhimurium*	*E. coli*	*V. cholerae*	*S. typhi*	*P. aeruginosa*	*K. pneumoniae*	*B. subtilis*	*S. aureus*
**23**	Br	200	100	200	50	50	200	200	50
**24**	Cl	200	50	100	200	100	200	50	50
Streptomycin	–	25	50	50	25	50	20	12.5	25

– not applicable

**Fig. 10:**
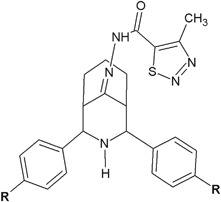
Methylthiadiazoles with significant activity against *Bacillus subtilis.* **R** = F (**26**); Br (**27**)

In another research, Kodisundaram et al. (2013) obtained a series of heterobicyclic methylthiadiazole hydrazones and investigated their activity in vitro against Gram-positive and Gram-negative bacteria. Two of the synthesized compounds, **26** and **27**, showed interesting activity, especially against *B. subtilis* (Fig. 10). Their activity against this bacterium (MIC = 6.25 μg/ml) was two times higher than the activity of streptomycin (MIC = 12.5 μg/ml), which was used as positive control. The activity of these compounds against *S. aureus* was good (MIC = 25–50 μg/ml). As for the Gram-negative bacteria, the synthesized compounds (**26**, **27**) showed two times better activity (MIC = 6.25 μg/ml) than streptomycin (MIC = 12.5 μg/ml) against *K. peneumoniae*, whereas against *P. aeruginosa*, compound **27** showed the activity equal to streptomycin (MIC = 12.5 μg/ml). The activity of the inhibition of the growth of *E. coli* was also two times higher (MIC = 12.5 μg/ml) in comparison with the control (MIC = 25 μg/ml) (Kodisundaram et al. 2013).

**Fig. 11:**
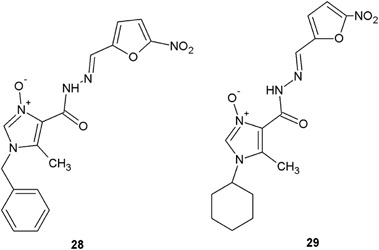
New imidazole derivatives containing hydrazide–hydrazone moiety

Pieczonka et al. (2013) synthesized a series of new imidazole derivatives containing hydrazide–hydrazone moiety and evaluated them for antibacterial activity against a panel of bacterial strains. Two of synthesized compounds (**28** and **29**) showed the best activity towards *Staphylococcus epidermidis* ATCC 12228 (MIC = 4 μg/ml) (Fig. 11). The activity of these compounds against this bacterium was two times higher than the activity of nitrofurantoin (MIC = 8 μg/ml). The MICs against the other bacterial strains like, *S. aureus* ATCC 6538, *S. aureus* ATCC 29213, and *S. aureus* ATCC 29213, were also low (MIC = 11–27 μg/ml) (Pieczonka et al. 2013).

**Fig. 12:**
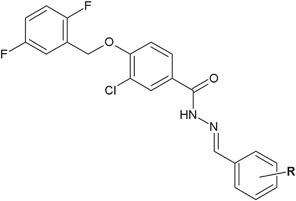
New derivatives of 2,5-difluorobenzoic acid with hydrazide–hydrazone moiety. **R** = 4-CF_3_ (**30**); 2-CF_3_ (**31**); 2,4-diF (**32**)

The infection caused by *P. aeruginosa* constitutes a severe problem, especially for the patients with weak immune system. In response to this, hydrazide–hydrazones of 2,5-difluorobenzoic acid were synthesized by Narisetty et al. (2013) (Fig. 12). Among a series of new derivatives, compound **30** showed better activity (ZOI = 21 mm) against *P. aeruginosa* MTCC 424 than ampicillin (ZOI = 20 mm). Two of other synthesized compounds (**31**, **32**) showed also very good activity (ZOI = 21–24 mm) against *E. coli* MTCC 443, *S. aureus* MTCC 96 and *Streptococcus pyogenes* MTCC 442. The activity of these compounds was higher than or comparable to the activity of ampicillin (ZOI = 19–22 mm) (Table 4) (Narisetty et al. 2013).

**Table 4:** Measured zones of the inhibition growth of hydrazide-hydrazones of 2,5-difluorobenzoic acid

No. of compound	R	Zone of inhibition growth (mm)			
		Gram-negative bacteria		Gram-positive bacteria	
		*E. coli* MTCC 443	*P. aeruginosa* MTCC 424	*S. aureus* MTCC 96	*S. pyogenes* MTCC 442
**30**	4-CF_3_	24	21	22	21
**31**	2-CF_3_	23	22	24	21
**32**	2,4-diF	24	21	23	21
Ampicillin	–	22	20	21	19

**Fig. 13:**
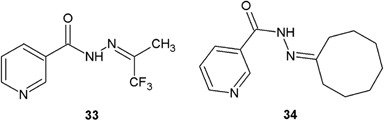
Novel hydrazide–hydrazones of nicotinic acid

Morjan et al. (2014) synthesized novel hydrazide–hydrazones of nicotinic acid by the condensation reaction of nicotinic acid hydrazide with various ketones (Fig. 13). The synthesized derivatives were screened in vitro for antibacterial activity against Gram-positive and Gram-negative bacteria. The results of antimicrobial assay revealed that the synthesized compounds had interesting antibacterial activity against Gram-negative strains. Especially compounds **33** and **34** showed very strong activity against *P. aeruginosa* (MIC = 0.22 and 0.19 μg/ml, respectively). The activity of these compounds against *K. pneumoniae* was also high (**33**: MIC = 3.12 μg/ml, **34**: MIC = 14.00 μg/ml). The inhibitory potency against Gram-positive bacteria *Streptococcus pneumoniae* (**33**: MIC = 3.12 μg/ml) and against *S. aureus* (**34**: MIC = 7.03 μg/ml) was also very significant (Morjan et al. 2014).

**Fig. 14:**
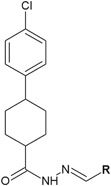
Novel hydrazide–hydrazone derivatives of 4-(4-chlorophenyl)cyclohexanecarboxylic acid as potential antibacterial agents. **R** = 2,4-diF-C_6_H_3_ (**35**); 2,6-diF-C_6_H_3_ (**36**); 3,4-diF-C_6_H_3_ (**37**)

Satyanarayana et al*.* (2014) synthesized novel hydrazide–hydrazone derivatives of 4-(4-chlorophenyl)cyclohexanecarboxylic acid as potential antibacterial agents (Fig. 14). Three of the obtained compounds (**35**, **36** and **37**) showed good antibacterial activity (MIC = 32–64 μg/ml) against a panel of four bacterial strains, including: *S. aureus*, *B. subtilis*, *E. coli*, and *P. aeruginosa* (Table 5). Unfortunately, the MIC values of the synthesized compounds were higher than the activity of the antibiotic used as control—ciprofloxacin (MIC = 5 μg/ml) (Satyanarayana et al*.* 2014).

**Table 5:** Results of in vitro screening of novel hydrazide-hydrazone derivatives of 4-(4-chlorophenyl)cyclohexanecarboxylic acid

No. of compound	R	MIC (μg/ml)			
		Gram-positive bacteria		Gram-negative bacteria	
		*S. aureus*	*B. subtilis*	*E. coli*	*P. aeruginosa*
**35**	2,4-diF-C_6_H_3_	64	64	32	32
**36**	2,6-diF-C_6_H_3_	64	64	32	32
**37**	3,4-diF-C_6_H_3_	64	64	32	32
Ciprofloxacin	–	5	5	5	5

– not applicable

**Fig. 15:**
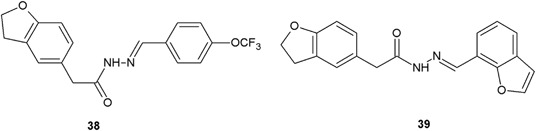
2-(2,3-Dihydrobenzofuran-5-yl)acetic acid derivatives with interesting antibacterial properties

2-(2,3-Dihydrobenzofuran-5-yl)acetic acid was used as a starting material for the synthesis of new hydrazide–hydrazone derivatives by Kaki et al. (2014). Among a series of synthesized derivatives, two compounds (**38** and **39**) possessed interesting antibacterial activity measured by the diameter of the zone of the inhibition growth against two Gram-negative (*E. coli* and *P. aeruginosa*) and two Gram-positive (*S. aureus* and *S. pyogenes*) bacterial strains (Fig. 15). Compounds (**38** and **39**) according to the antimicrobial activity assays, had similar values of inhibition zone (ZOI = 19–22 mm) as ampicillin, which was used as positive control (ZOI = 19–22 mm) (Kaki et al. 2014).

**Fig. 16:**
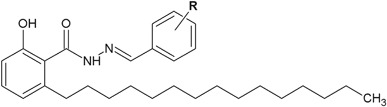
New anacardic acid hydrazide derivatives. **R** = 3,4-diOCH_3_ (**40**); 3,4,5-triOCH_3_ (**41**); 4-SO_2_CH_3_ (**42**)

In the research conducted by Rambabu et al*.* (2015), new anacardic acid hydrazone derivatives were synthesized and subjected to antibacterial screening. The obtained compounds were tested against two Gram-negative (*P. aeruginosa* and *E. coli*) and two Gram-positive (*S. aureus* and *S. pyogenes*) bacterial strains (Fig. 16). The antibacterial activity was assessed on the basis of the measurement of the zone of inhibition growth. Antimicrobial assays revealed that three of the obtained derivatives (**40**, **41** and **42**) had better antibacterial activity (ZOI = 20–24 mm) than the activity of ampicillin, because their zones of inhibition were larger than the control antibiotic (ZOI = 18–20 mm) (Table 6) (Rambabu et al*.* 2015).

**Table 6:** Zone of inhibition growth of tested anacardic acid hydrazide derivatives

No. of compound	R	Zone of inhibition growth (mm)					
		Gram-negative bacteria		Gram-positive bacteria		Fungi	
		*P. aeruginosa*	*E. coli*	*S. aureus*	*S. pyogenes*	*C. albicans*	*A. niger*
**40**	3,4-diOCH_3_	24	23	21	22	11	12
**41**	3,4,5-triOCH_3_	22	22	20	21	18	23
**42**	4-SO_2_CH_3_	23	22	20	20	18	20
Ampicillin	–	20	20	18	19	–	–
Griseofulvin	–	–	–	–	–	24	28

– not applicable

**Fig. 17:**
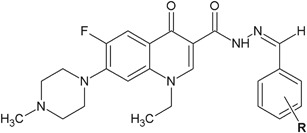
Novel pefloxacin derivatives with interesting antibacterial activity. **R** = 2,6-diCl (**43**); 4-NO_2_ (**44**); 3,4,5-triOCH_3_ (**45**); 5-Br-2-OH (**46**); 2,5-diOCH_3_ (**47**); 3-OH (**48**)

Tejeswara et al*.* (2016) synthesized a series of novel pefloxacin derivatives and tested them for their in vitro antibacterial activity against four Gram-positive bacterial strains and two Gram-negative bacterial strains by the agar well diffusion method (Fig. 17). On the basis of the measurement of the zone of inhibition growth, antibacterial activity assay revealed that compounds **46** and **47** (ZOI = 24 and 26 mm, respectively) had higher antibacterial activity compared to ciprofloxacin (ZOI = 23 mm) against *Bacillus sphaericus*. Against other *Bacillus* strain, *B. subtilis* compound **47** had a larger zone of inhibition (ZOI = 23 mm) than the reference substance (ZOI = 22 mm). Compound **47** also displayed higher activity against *Pseudomonas aeruginosa* (ZOI = 25 mm) than ciprofloxacin (ZOI = 21 mm) (Table 7) (Tejeswara et al*.* 2016).

**Table 7:** Measured zones of inhibition growth of pefloxacin derivatives

Compound	Zone of inhibition growth (mm)					
	Gram-positive bacteria				Gram-negative bacteria	
	*S. aureus*	*B. sphaericus*	*B. subtilis*	*M. luteus*	*P. aeruginosa*	*P. vulgaris*
**43**	20	15	16	30	15	10
**44**	18	16	15	20	14	11
**45**	19	22	17	15	21	12
**46**	19	24	15	12	14	16
**47**	18	26	23	15	25	17
**48**	21	21	16	14	14	15
Ciprofloxacin	20	23	22	18	21	17

**Fig. 18:**
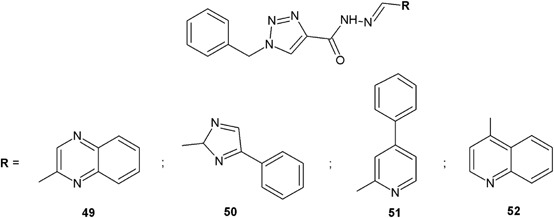
*N*-substituted-1-benzyl-1*H*-1,2,3-triazole-carbohydrazide derivatives with potential antibacterial activity

Novel 1,2,3-triazole carbohydrazide derivatives synthesized by Sreedhar et al*.* (2016) were tested against four bacterial strains (Fig. 18). The measured zones of growth inhibition for the obtained compounds were similar to those of ciprofoxacin used as control. In the case of compound **50**, its zone of inhibition against *S. aureus* MTCC 96 (ZOI = 23 mm) was larger than for the reference compound (ZOI = 22 mm). A similar situation appeared for compounds **49** and **52**, their zones of inhibition (ZOI = 23 mm) were larger than for ciprofloxacin (ZOI = 22 mm) against *S. pyogenes* MTCC 442 (Table 8) (Sreedhar et al*.* 2016).

**Table 8:** The values of the zone of the inhibition growth of 1,2,3-triazole-carbohydrazide derivatives

No. of compound	Zone of inhibition growth (mm)			
	Gram-positive bacteria		Gram-negative bacteria	
	*S. aureus* MTCC 96	*S. pyogenes* MTCC 442	*E. coli* MTCC 443	*P. aeruginosa* MTCC 424
**49**	21	23	27	26
**50**	23	22	26	25
**51**	22	21	25	26
**52**	22	23	24	23
Ciprofloxacin	22	22	28	27

**Fig. 19:**
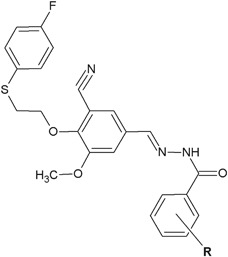
Benzohydrazide derivatives with interesting antibacterial activity. **R** = OH (**53**); 3,4,5-triOCH_3_ (**54**)

Dommati et al. (2016) obtained novel benzohydrazide derivatives and evaluated them for in vitro antibacterial activity (Fig. 19). The highest activity, but weaker than control streptomicin, was shown by compounds **53** and **54** against Gram-negative bacteria: *E. coli* MTCC 2692 and *P. aeruginosa* MTCC 2453 (ZOI = 5–13 mm), and Gram-positive bacteria: *S. aureus* MTCC 902 and *B. subtilis* MTCC 441 (ZOI = 18–21 mm) (Dommati et al. 2016).

## Antitubercular activity

Despite the fact that tuberculosis is a curable and treatable disease, it still remains a major cause of death and illness (WHO 2010), which confirms that seeking and developing new antitubercular agents is needed. A survey of scientific literature reveals that several hydrazide–hydrazones synthesized during last 6 years possess interesting antitubercular activity (Unissa et al*.* 2016). Additionally, it is worth to mention that according to the most recent article published by John et al. (2016) the 2-hydroxy-1-naphthaldehyde isonicotinoyl hydrazone may act as a novel inhibitor of methionine aminopeptidases from *Mycobacterium tuberculosis.*

**Fig. 20:**
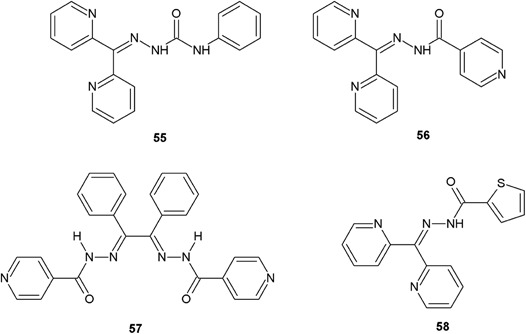
Hydrazide–hydrazones with interesting antitubercular activity

Among the series of hydrazide–hydrazones synthesized by Pavan et al. (2010), the four compounds (**55**, **56**, **57**, **58**) showed especially high activity towards *M. tuberculosis* (MIC = 1.5–12.5 μg/ml) (Fig. 20) (Pavan et al. 2010).

**Fig. 21:**
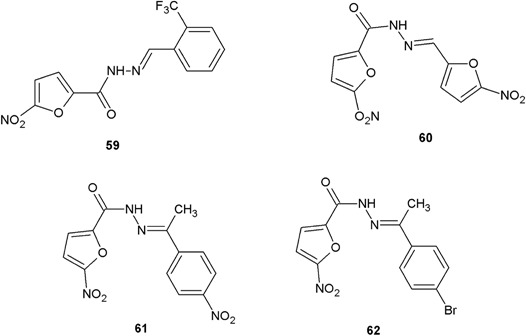
New 5-nitro-2-furoic acid hydrazide derivatives with potent in vitro antitubercular activity

Sriram et al*.* (2010) tested for antitubercular activity a new series of 5-nitro-2-furoic acid hydrazones. New compounds were obtained by the condensation reaction of 5-nitro-2-furoic hydrazide with appropriate aldehydes and ketones (Fig. 21). In vitro screening of the obtained compounds revealed potent antitubercular activity of synthesized derivatives (**59**–**62**). Especially compounds **59** and **60** showed very good activity (MIC = 4.76 and 2.65 µM, respectively) in comparison with isoniazid (MIC = 0.72 µM) and rifampicin (MIC = 0.48 µM) (Sriram et al*.* 2010).

**Fig. 22:**
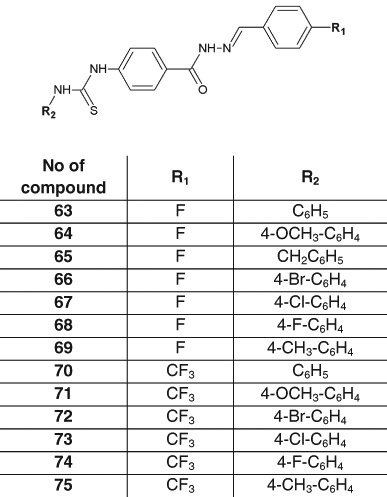
Novel thioureas with activity against *Mycobacterium tuberculosis* H37Rv

Novel thioureas containing hydrazide–hydrazone moiety were synthesized and tested against *M. tuberculosis* H37Rv (ATCC 27294) by Çıkla et al. (2010) (Fig. 22). In the antitubercular assays, these compounds showed lower activity (MIC > 6.25 μg/ml) than rifampicin used as a reference substance (Çıkla et al. 2010).

**Fig. 23:**
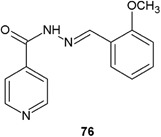
Hydrazide–hydrazone obtained from isonicotinic hydrazide with in vitro activity against *M. tuberculosis* isoniazid-susceptible strain

Coelho et al.’s study (2012) evaluated the in vitro antibacterial activity of 23 hydrazide–hydrazones obtained from isonicotinic hydrazide against one *M. tuberculosis* isoniazid-susceptible strain and three isoniazid-resistant *M. tuberculosis* clinical isolates (Fig. 23). Interestingly, 13 derivatives showed good activity against isoniazid-resistant strains. The best activity (better than that of isoniazid against isoniazid-susceptible strain) was shown by compound **76** (MIC = 0.98 μg/ml) (Coelho et al. 2012).

**Fig. 24:**
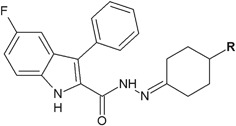
Indole derivatives containing hydrazide–hydrazone scaffold with antitubercular activity. **R** = H (**77**); CH_3_ (**78**); C_2_H_5_ (**79**); C_3_H_7_ (**80**); C_6_H_5_ (**81**)

Cihan-Üstündağ and Çapan (2012) evaluated a series of indole derivatives containing hydrazide–hydrazone scaffold for in vitro antitubercular activity (Fig. 24). Antimycobacterial activity was tested against *M. tuberculosis* H37Rv ATCC 27294 with the use of rifampicine as a control. Unfortunately the synthesized compounds showed weaker activity (MIC > 6.25 μg/ml) than the reference substance (MIC = 0.125 μg/ml) (Cihan-Üstündağ and Çapan 2012).

**Fig. 25:**
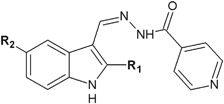
Novel indole derivatives with significant antitubercular activity. **82**: **R**_**1**_ = COOH, **R**_**2**_ = H; **83**: **R**_**1**_ = COOC_2_H_5_, **R**_**2**_ = H; **84**: **R**_**1**_ = COOC_2_H_5_, **R**_**2**_ = Cl; **85**: **R**_**1**_ = COOC_2_H_5_, **R**_**2**_ = CH_3_

Velezheva et al*.* (2016) designed and synthesized a series indole-pyridine derived hydrazide–hydrazones and evaluated them against two *M. tuberculosis* strains (H37Rv and CN-40) (Fig. 25). Based on the obtained results, hydrazide–hydrazone derivative **85** appeared to be the most potent among examined compounds (MIC=0.05 µg/ml) against the *M. tuberculosis* H37Rv strain. Its activity was equal to that of isoniazid used as positive control in this assay. Besides, this compound **85**, unlike isoniazid, showed significant activity against isoniazid-resistant *M. tuberculosis* CN-40 strain. Other synthesized derivatives also displayed high antitubercular activity (Table 9) (Velezheva et al*.* 2016).

**Table 9:** MIC values of indole derivatives with hydrazide–hydrazone moieties

No. of compound	MIC (μg/ml)	
	*M. tuberculosis* H37Rv	*M. tuberculosis* CN-40
**82**	0.1	10
**83**	0.15	2–5
**84**	0.1	2–5
**85**	0.05	2–5

## Antifungal activity

Fungal infections still remain a serious problem, even though there are many available medicines on the market (Lewis 2011). In this section, I will present several examples of hydrazide–hydrazones which possess significant antifungal activity.

Benzimidazole derivatives bearing hydrazone moiety (Özkay et al. 2010) were also screened for antifungal activity against three species of yeasts: *Candida albicans*, *Candida glabrata*, and *Candida tropicalis*. The activity of compounds **1** and **2** against these fungi was good (MIC = 50–100 μg/ml). In the case of *C. tropicalis*, the MICs of the synthesized compounds (MIC = 50 μg/ml) were equal to the MIC of ketoconazole used as control (MIC = 50 μg/ml) (Özkay et al. 2010).

Similar research was performed by Kumar et al. (2011a). New hydrazide–hydrazones of 4-chlorophenylsulfonyl acid were synthesized and tested for antifungal activity on the basis of the measurement of the zone of inhibition growth against *C. albicans* and *Aspergillus niger*. Three of the synthesized compounds (**8**, **9** and **10**) showed promising antifungal activity compared with the clotrimazole, which was used as positive control. In the case of compound **8**, the antifungal activity (ZOI = 31 mm) was greater than the activity of clotrimazole (ZOI = 30 mm) against *C. albicans* (Kumar et al. 2011a).

The compounds synthesized by Shirinzadeh et al*.* (2011) (**12**, **13**) were subjected to antifungal assays against *C. albicans*. The revealed antifungal activity was strong for both compounds (**12**: MIC = 6.25 μg/ml and **13**: 12.5 μg/ml, respectively), but weaker than for fluconazole (MIC = 0.78 μg/ml) (Shirinzadeh et al. 2011).

New hydrazide–hydrazone derivatives (**23**, **24** and **25**) (Xaiver et al*.* 2012) were tested against a panel of five fungi strains including *C. albicans*, *Fusarium oxysporum*, *Aspergillus flavus*, *A. niger*, and *Cryptococcus neoformans*. The measured MIC parameters against these fungi (MIC = 50–200 μg/ml) were much higher than the activity of amphotericin B used as control substance (MIC = 25–50 μg/ml), and the activity of these compounds can only be assessed as good against *A. niger* (MIC = 50–100 μg/ml), and as moderate against other fungi (MIC = 100–200 μg/ml) (Table 10) (Xaiver et al*.* 2012).

**Table 10:** In vitro antifungal data of hydrazide–hydrazone derivatives of 4-aminobenzoic acid hydrazide

No. of compound	R	MIC (μg/ml)				
		*C. albicans*	*F. oxysporum*	*A. flavus*	*A. niger*	*C. neoformans*
**23**	Br	200	100	100	50	100
**24**	Cl	100	200	100	50	200
**25**	OCH_3_	200	100	50	100	200
Amphotericin B	–	25	25	50	50	25

– not applicable

Novel heterobicyclic methylthiadiazole hydrazones synthesized by Kodisundaram et al. (2013) were also tested for antifungal activity against *Aspergillus* spp. and *C. albicans*. The activity of compounds **26** and **27** against *A. flavus* and *A. niger* was two times higher (MIC = 12.5 μg/ml) than the antifungal activity of fluconazole (MIC = 25 μg/ml). The MICs against *C. albicans* for synthesized compounds were also two times higher (MIC = 6.25 μg/ml) than the MIC value of fluconazole (12.5 μg/ml) (Kodisundaram et al. 2013).

New anacardic acid hydrazone derivatives were subjected to in vitro antifungal assays against *C. albicans* and *A. niger* (Rambabu et al*.* 2015). Three of the synthesized compounds (**40**, **41** and **42**) showed good to moderate antifungal activity (ZOI = 11–20 mm) based on the measurement of the zone of inhibition growth in comparison with the gryseofulvin used as control (ZOI = 24–28 mm) (Rambabu et al*.* 2015).

**Fig. 26:**
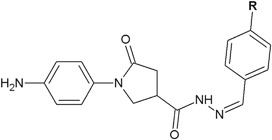
Pyrrolidinones with hydrazone moieties with antifungal activity. **R**=N(CH_3_)_2_ (**86**); Cl (**87**)

Pyrrolidinones with hydrazone moieties synthesized by Rutkauskas et al*.* (2013) showed strong (**86**) or good (**87**) activity against *Candida tenuis* (**86**: MIC = 15.6 µg/ml and **87**: 31.2 µg/ml, respectively) (Fig. 26), whereas the activity against *A. niger* was lower (MIC = 500 µg/ml) (Rutkauskas et al*.* 2013).

**Fig. 27:**
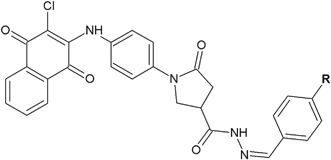
Hydrazide–hydrazones with significant antifungal activity against *Candida tenuis* and *Aspergillus niger.* **R**=N(CH_3_)_2_ (**88**); Cl (**89**)

In the case of compounds **88** and **89** the antifungal activity against *C. tenuis* and *A. niger* was much better (Fig. 27). Compounds **88** and **89** against *C. tenuis* showed the MIC values of 1.9 and 0.9 µg/ml, respectively. Compound **88** displayed strong activity (MIC = 3.9 µg/ml) against *A. niger* and compound **89** showed moderate activity (MIC = 125 µg/ml) (Rutkauskas et al*.* 2013).

**Fig. 28:**
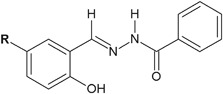
Hydrazide–hydrazones of benzoic acid with antifungal activity. **R** = H (**90**); CH_3_ (**91**); OCH_3_ (**92**); Cl (**93**)

Hydrazide–hydrazones of benzoic acid synthesized by Backes et al. (2014) showed interesting antifungal activity against *Candida* spp. (Fig. 28). The activity of compounds **90**–**93** was very strong (MIC_80_ = 0.5–4 µg/ml) against *C. albicans*. *C. glabrata* was also very sensitive to the obtained benzoic acid derivatives (MIC_80_ = 0.5–1.0 µg/ml) (Table 11) (Backes et al. 2014).

**Table 11:** MIC_80_ values of hydrazide–hydrazones of benzoic acid

No. of compound	R	MIC_80_ (μg/ml)	
		*C. albicans*	*C. glabrata*
**90**	H	2	1
**91**	CH_3_	1	1
**92**	CH_3_O	4	0.5
**93**	Cl	0.5	0.5

**Fig. 29:**
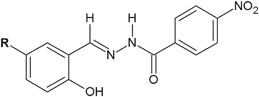
Hydrazide–hydrazones of 4-nitrobenzoic acid with the antifungal activity. **R** = H (**94**); CH_3_ (**95**)

In the case of hydrazide–hydrazones of 4-nitrobenzoic acid, the antifungal activity against the above mentioned fungi strains was even better (Fig. 29). The MIC_80_ parameters for compounds **94** and **95** were 0.5 µg/ml against *C. albicans*, whereas for compound **95** it was even 0.125 µg/ml against *C. glabrata*. The MIC_80_ value for compound **94** against *C. glabrata* was also very low (MIC_80_ = 0.5 µg/ml) (Backes et al. 2014).

**Fig. 30:**
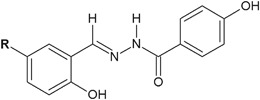
Hydrazide–hydrazone derivatives of 4-hydroxybenzoic acid with activity against *Candida glabrata*. **R** = H (**96**); CH_3_ (**97**)

Additionally, compounds **96** and **97** as hydrazide–hydrazone derivatives of 4-hydroxybenzoic acid displayed activity only against *C. glabrata* (Fig. 30). The MIC_80_ values against this fungus were 4 and 1 µg/ml for compounds **96** and **97**, respectively (Backes et al. 2014).

## Conclusions

In conclusion, this paper gives an overview of the antibacterial, antitubercular and antifungal properties of hydrazide-hydrazone derivatives. As presented in this study hydrazide–hydrazone moiety may be found and incorporated in various bioactive molecules. Thus this paper appears to be important for further development of hydrazide–hydrazones as potential antimicrobial agents.

## Abbreviations

MIC: Minimal inhibitory concentration
ZOI: Zone of inhibition
*A. flavus*: *Aspergillus flavus*
*A. niger*: *Aspergillus niger*
*B. megaterium*: *Bacillus megaterium*
*B. sphaericus*: *Bacillus sphaericus*
*B. subtilis*: *Bacillus subtilis*
*C. albicans*: *Candida albicans*
*C. glabrata*: *Candida glabrata*
*C. tropicalis*: *Candida tropicalis*
*C. tenuis*: *Candida tenuis*
*C. neoformans*: *Cryptococcus neoformans*
*E. aerogenes*: *Enterobacter aerogenes*
*E. faecalis*: *Enterococcus faecalis*
*E. coli*: *Escherichia coli*
*F. oxysporum*: *Fusarium oxysporum*
*K. pneumoniae*: *Klebsiella pneumoniae*
*L. monocytogenes*: *Listeria monocytogenes*
*M. luteus*: *Micrococcus luteus*
*M. tuberculosis*: *Mycobacterium tuberculosis*
*P. mirabilis*: *Proteus mirabilis*
*P. vulgaris*: *Proteus vulgaris*
*P. aeruginosa*: *Pseudomonas aeruginosa*
*P. picketti*: *Pseudomonas picketti*
*S. enterica*: *Salmonella enterica*
*S. typhi*: *Salmonella typhi*
*S. typhimurium*: *Salmonella typhimurium*
*S. aureus*: *Staphylococcus aureus*
*S. epidermidis*: *Staphylococcus epidermidis*
*S. pneumoniae*: *Streptococcus pneumoniae*
*V. cholerae*: *Vibrio cholerae*
